# Design and Factorial Optimization of Curcumin and Resveratrol Co-Loaded Lipid Nanocarriers for Topical Delivery

**DOI:** 10.3390/pharmaceutics18010109

**Published:** 2026-01-15

**Authors:** Daniela Pastorim Vaiss, Débora Cristine Chrisostomo Dias, Virginia Campello Yurgel, Fernanda Beatriz Venturi Araujo, Ledilege Cucco Porto, Janaina Fernandes de Medeiros Burkert, Marcelo Augusto Germani Marinho, Daza de Moraes Vaz Batista Filgueira, Cristiana Lima Dora

**Affiliations:** 1 Programa de Pós-Graduação em Ciências da Saúde, Universidade Federal do Rio Grande-FURG, Rio Grande 96203-900, RS, Brazil; debichrisostomo@gmail.com; 2 Laboratório de Nanotecnologia, Universidade Federal do Rio Grande-FURG, Rio Grande 96203-900, RS, Brazil; virginia.yurgel@gmail.com (V.C.Y.); fernandabeatriz237@gmail.com (F.B.V.A.); 3 ALKBIO Cosmetic Technology, Florianopolis 88030-902, SC, Brazil; ledilege@alkbio.com.br; 4 Programa de Pós-Graduação em Engenharia e Ciências de Alimentos, Universidade Federal do Rio Grande-FURG, Rio Grande 96203-900, RS, Brazil; jfmb@furg.br; 5 Laboratório de Cultura Celular, Instituto de Ciências Biológicas, Universidade Federal do Rio Grande-FURG, Rio Grande 96203-900, RS, Brazil; marceloaugustomarinho@gmail.com (M.A.G.M.); dazafil@gmail.com (D.d.M.V.B.F.); 6 Programa de Pós-Graduação em Ciências Fisiológicas, Universidade Federal do Rio Grande-FURG, Rio Grande 96203-900, RS, Brazil

**Keywords:** factorial design, dermatoses, antioxidants, natural compounds

## Abstract

**Background**: Nanotechnology provides innovative strategies to enhance drug delivery and therapeutic efficacy through advanced nanocarrier systems. **Objectives**: This study aimed to develop and optimize a nanostructured lipid carrier (NLC) co-encapsulating curcumin (CUR) and resveratrol (RESV) using a fractional factorial design to develop a topical formulation with antioxidant and anti-inflammatory properties. **Methods:** NLCs were produced via hot emulsification followed by high-pressure homogenization, and their physicochemical characteristics, drug content, stability, release profile, antioxidant activity, skin delivery, and cellular compatibility were evaluated. **Results:** The optimized formulation exhibited an average particle size of approximately 300 nm, a polydispersity index below 0.3, and high drug loading for both compounds. Stability studies over 90 days revealed no significant changes in physicochemical parameters, confirming the formulation’s robustness. In vitro release assays demonstrated sustained release of both actives, with 58.6 ± 2.9% of CUR and 97 ± 3% of RESV released after 72 h. Antioxidant activity, assessed by the DPPH and ABTS assays, showed concentration-dependent radical-scavenging effects, indicating antioxidant potential. Skin permeation/retention experiments using porcine skin showed enhanced retention of CUR and RESV within the tissue, with no detectable permeation, indicating suitability for topical delivery. In addition, in vitro cell assays using human keratinocytes showed concentration-dependent responses and acceptable cellular compatibility. **Conclusions:** Overall, this study demonstrates the successful application of nanotechnology and experimental design to develop stable and efficient lipid-based nanocarriers containing natural polyphenol for topical therapy targeting oxidative and inflammatory skin disorders.

## 1. Introduction

Nanotechnology is a multidisciplinary science that studies materials at the nanoscale. In recent years, advances in nanotechnology have been evident across various areas due to the properties enabled by material miniaturization [1,2].

To better contextualize their relevance to drug delivery, nanocarriers are used in pharmacology to enhance the therapeutic performance of various active compounds. Due to their size, nanocarriers can be used to improve the pharmacokinetic profile of active compounds, enable site-specific delivery at therapeutic sites, prolong the release kinetics of active compounds, reduce the number of doses required, enable local applications, and avoid systemic effects, among other uses [3,4].

Various types of nanocarriers can be developed and classified based on their composition, shape, size, and physicochemical properties. However, lipid-based systems are particularly relevant for dermatological applications due to their skin permeability and biocompatibility [4]. In this context, to achieve optimal therapeutic efficacy, formulation development must simultaneously balance multiple interdependent parameters, such as particle size, drug loading capacity, encapsulation efficiency, and overall stability. In addition, when multiple bioactive compounds are incorporated into the same system, the formulation challenge increases due to their distinct physicochemical properties [2,5].

Building upon these formulation challenges, despite the availability of conventional topical treatments for inflammatory skin diseases such as psoriasis, atopic dermatitis, and leprosy, these approaches face significant limitations that compromise therapeutic efficacy and patient adherence. The stratum corneum acts as a formidable barrier, resulting in poor drug penetration and low bioavailability of active ingredients, often requiring high concentrations that can cause skin irritation, atrophy, or allergic reactions [6,7,8]. Moreover, many formulations exhibit instability under physiological conditions, undergo rapid degradation, or exhibit inconsistent release profiles, necessitating frequent administration, which exacerbates nonadherence and increases the risk of systemic absorption and associated side effects, such as immunosuppression or hormonal imbalances [9,10]. These challenges are particularly pronounced in chronic conditions, where sustained anti-inflammatory and antioxidant effects are essential to mitigate oxidative stress and cytokine storms without long-term toxicity. In this regard, nanotechnology-based systems offer a promising alternative by enhancing targeted delivery, stability, and controlled release, addressing the unmet needs of conventional therapies [11,12].

Given these therapeutic and technological limitations, this underscores the complexity of experimental formulation development, as any change can affect the nanocarrier’s final properties. The need for reproducible results compounds this. For these purposes, experimental design tools can be applied, in which statistical methods aid in understanding the responses generated by the system [3,13]. Multivariate factorial design differs fundamentally from conventional “one-factor-at-a-time” approaches: it reveals synergistic interactions between formulation variables, requires fewer experiments, and generates statistically robust conclusions about complex systems. This approach enables identification of the most efficient combination of bioactive compounds and excipients in the formulation, rather than through trial and error [5,13,14].

In parallel with methodological advances, studies investigate the biological activity of natural compounds and their use, indicating their efficacy and safety for long-term therapies. However, a persistent challenge in studies of natural molecules is the difficulty of developing efficient, reproducible formulations that better leverage the therapeutic advantages of these compounds. Based on these facts, lipid nanocarrier development can be explored to formulate a formulation containing natural bioactive compounds of interest for enhanced therapeutic efficacy [15,16].

Resveratrol (RESV) is a naturally occurring polyphenolic compound belonging to the polyphenol family. It has two stereoisomers, cis and trans, and appears in various botanical sources, including grapes and other red fruits [16,17]. It demonstrates multiple bioactive effects, such as modulation of inflammatory pathways, antioxidant activity and inhibition of microbial growth, which are the main properties highlighted in studies supporting its dermatological use [18]. Another natural compound that has been extensively studied, including for dermatological applications, is curcumin (CUR), extracted from turmeric, which also belongs to the polyphenol family and has some pharmacological activities similar to those of RESV. However, among its medicinal activities, it may have different, complementary mechanisms of action and varied pharmacokinetics [19,20].

Among the activities described in the literature for these compounds, antioxidant activity is particularly prominent for dermatological applications. The skin, as the largest organ of defense, is constantly exposed to endogenous and environmental factors that trigger the production of reactive oxygen species (ROS) and nitrogen species (RNS). When their generation exceeds the capacity of enzymatic and non-enzymatic antioxidant systems, such as superoxide dismutase (SOD), catalase (CAT), and glutathione peroxidase (GPx), oxidative stress is triggered, culminating in lipid, protein, and deoxyribonucleic acid (DNA) impairment [21,22,23]. This redox imbalance weakens the skin barrier and activates inflammatory signaling cascades, notably Nuclear Factor kappa-light-chain-enhancer of activated B cells (NF-κB) and NOD-like receptor family pyrin domain containing 3 (NLRP3), which drive the release of pro-inflammatory cytokines and further propagate oxidative damage [24,25,26]. In chronic dermatoses such as psoriasis, atopic dermatitis, and infectious skin diseases, elevated oxidative stress biomarkers and reduced endogenous antioxidant capacity have been consistently reported, and these biomarkers correlate with disease severity and impaired skin homeostasis [27,28,29].

The simultaneous co-administration of natural bioactive compounds in a single formulation has been widely investigated, often yielding synergistic or additive effects depending on the components’ molecular interactions and biological responses. The combination of distinct phytochemicals may allow the use of lower individual concentrations while maintaining or even enhancing therapeutic efficacy compared to the isolated compounds. Moreover, this strategy can mitigate concentration-dependent adverse or toxic effects commonly associated with higher doses of individual actives [15,19,30,31].

In this context, the present study aimed to develop and optimize a nanostructured lipid carrier (NLC) co-loading CUR and RESV using a fractional factorial design to evaluate the influence of formulation variables on key physicochemical attributes. The optimized formulation was subsequently characterized for stability, in vitro release behavior, antioxidant activity, ex vivo skin permeation and retention, and in vitro cellular compatibility, providing a structured assessment of a lipid-based nanocarrier platform for topical therapy targeting oxidative and inflammatory skin disorders.

## 2. Materials and Methods

Curcumin (≥65%, HPLC), DPPH (2,2-difenil-1-picril-hidrazila), Ácido 2,2′-azino-bis(3-etilbenzotiazolina-6-sulfônico), castor oil, sorbitan monooleate (Span^®^ 80), oily vitamin E, Dulbecco’s Modified Eagle Medium (DMEM), 3-(4,5-Dimethyl-2-thiazolyl)-2,5-diphenyl-2H-tetrazolium bromide (MTT), and sulforhodamine-B (SRB) were purchased from Sigma–Aldrich (St. Louis, MO, USA). Fetal bovine serum (FBS), penicillin/streptomycin, and amphotericin B were obtained from Gibco, Invitrogen (São Paulo, SP, Brazil). Resveratrol (≥98%, HPLC) was obtained from ISOBIO (Porto Alegre, RS, Brazil). Shea butter and coconut oil were supplied by Atika Cosméticos (São Paulo, Brazil). Sucrose distearate (SP30-C) was provided by Sisterna (Distributed by Galena, Campinas, SP, Brazil), and heptyl glucoside (Sepiclear G7) by Seppic Brazil (São Paulo, SP, Brazil). They were obtained from Embacaps (São Paulo, SP, Brazil). Benzyl alcohol, sodium benzoate, and polysorbate were used as preservatives. Acetonitrile and methanol (HPLC grade) were purchased from Sigma Aldrich, and Milli-Q^®^ ultrapure water was produced using a Millipore purification system (Burlington, MA, USA).

### 2.1. Factorial Planning Approach

In this study, a fractional factorial design was used to systematically investigate the effects of formulation variables on the physicochemical properties of NLCs. The 2^(4−1)^ design was selected as an efficient screening strategy capable of identifying both main effects and two-factor interactions while reducing the total number of experimental runs, which is particularly advantageous in multicomponent lipid systems [32,33,34]. The four formulation factors included in the design were selected based on their physicochemical properties, such as polarity, melting behavior, viscosity, and solubilization capacity, which are known to affect critical quality attributes of NLC, including particle size, polydispersity, and bioactive loading [35,36,37]. The maximum and minimum limits of each independent variable were established based on the results obtained in the preliminary investigation. Their minimum/maximum ranges were defined after preliminary tests evaluating the solubility of bioactive compounds, the melting behavior, and the processability by high-pressure homogenization [38,39,40].

The experimental design adopted was 2^(4−1)^, in which the independent variables corresponded to the lipids and one of the surfactants used in the oil phase of the nanocarrier. The dependent variables analyzed were particle size (Y1), polydispersity index (Y2), and bioactive content of curcumin (Y3) and resveratrol (Y4), as described in Table 1. Three center-point replicates were incorporated to estimate pure experimental error and assess potential curvature in the response, following recommended practices for fractional factorial screening designs [33,34]. This model allowed us to evaluate not only the main effects of each factor, but also interactions of up to four factors, using a reduced number of experiments (n = 11).

### 2.2. Development of Nanocarriers Containing CUR and RESV

The NLCs were obtained through thermal emulsification and subsequent high-pressure homogenization. For this purpose, two distinct phases were prepared: the aqueous phase, composed of milli-Q^®^ water and surfactant, and the oil phase, composed of oils, wax, surfactant, and the bioactive compounds of interest in this study, as shown in Table 2 and Table S2 (Supplementary Material). Briefly, the lipid phase was maintained at 80 °C under magnetic agitation (1500 rpm) for 30 min, while the aqueous phase was rapidly heated to 80 °C for 2 min and gently stirred at 300 rpm. At the end of the heating steps, the aqueous phase was added to the oil phase, and heating and stirring were maintained for 2 min. Next, the emulsion was stirred at 14,500 rpm for 3 min, and the process was completed by applying 11 cycles of 24 s (4 min and 24 s) of high-pressure homogenization at 10,000 psi. Finally, the formulation was stored at room temperature (22–24 °C) for subsequent steps.

### 2.3. Determination of Particle Size and Zeta Potential

The mean hydrodynamic diameter, polydispersity index (PDI), and zeta potential of the NLCs were evaluated using Dynamic Light Scattering (DLS) and Laser Doppler Anemometry, with analyses conducted on a Zetasizer Nano Series instrument (Malvern Panalytical, Malvern, UK). Measurements were carried out at 25 °C after diluting the formulations in distilled water at a ratio of 1:1000 (approximately 1 μL of nanocarrier per mL). Each particle size assessment was recorded over a 300 s acquisition period at a detection angle of 90°. The hydrodynamic radius was calculated based on the Stokes–Einstein equation (Equation (1)):

R = kBT/6πƞD(1)

where B represents Boltzmann’s constant (J/K), T is the absolute temperature (K), D is the diffusion coefficient, and η corresponds to the viscosity of the medium, which in this case is water (0.89 cP at 25 °C).

For the zeta potential analysis, the samples were introduced into the electrophoretic cell, and an alternating electric field of ±150 mV was applied. The zeta potential values were obtained from the average electrophoretic mobility using Smoluchowski’s equation.

#### Morphological Evaluation

The morphology of the NLCs was examined by transmission electron microscopy (TEM) using a JEOL 1400 instrument (Indianapolis, IN, USA). For sample preparation, the dispersion was diluted at a 1:500 ratio in Milli-Q^®^ water, and a small aliquot was placed on a carbon-coated copper grid (200 mesh; Koch Instrumentos Científicos, São Paulo, SP, Brazil). Subsequently, 20 µL of a 2% *w*/*v* uranyl acetate solution was applied for negative staining. Images were obtained at an accelerating voltage of 80 kV, with magnifications of 50,000× and 100,000×.

### 2.4. Determination of CUR and RESV Concentrations in NLCs

#### 2.4.1. Instruments and Analytical Conditions

For the simultaneous quantification of CUR and RESV in the NLCs, high performance liquid chromatography (HPLC) was carried out following the procedure reported by Nasr and Abdel Rahman (2019) [41]. The analyses were conducted on a Perkin Elmer Flexar system (Perkin Elmer Inc., Shelton, CT, USA) equipped with a quaternary pump, photodiode array detector, and autosampler. Chromatographic separation was achieved on a C18 column (150 × 4.6 mm, 5 µm; Zorbax ODS, Agilent Technologies, Wilmington, DE, USA) under isocratic conditions. The mobile phase consisted of Eluent A (aqueous phosphoric acid 1% *w*/*v*, adjusted to pH 2.6) and Eluent B (acetonitrile), delivered by the instrument at a 50:50 proportion with a flow rate of 1.0 mL/min. Detection was performed at 305 nm for RESV and 425 nm for CUR.

Calibration curves were prepared for each compound to assess linearity, using standards at concentrations of 0.25, 0.5, 1.0, 2.0, 4.0, 6.0, 8.0, and 10.0 µg/mL. Stock solutions at 1 mg/mL were prepared by dissolving the compounds in methanol, and calibration points were obtained by serial dilution. To assess accuracy and precision, three concentrations (0.5, 4.0, and 10.0 µg/mL) were analyzed in triplicate on the same day and on subsequent days, using freshly prepared standards. To evaluate potential interference from the nanocarrier, 10 µL of blank nanocarrier (without bioactive compounds) was added to the standards. The calibration curves for both analytes showed linearity within the concentration range of 0.25–10 µg/mL, with correlation coefficients of 0.99. The LOD and LOQ values were 0.05 and 0.15 µg/mL for CUR and 0.08 and 0.23 µg/mL for RESV. Method specificity was verified through the separate analysis of unloaded colloidal formulations (see Supplementary Material Table S1).

#### 2.4.2. Determination of CUR and RESV Content, Recovery and Encapsulation Efficiency

For HPLC quantification, a defined amount of the nanocarrier formulation was placed into a 10.0 mL volumetric flask to obtain the test solution, and the volume was completed with methanol. The CUR and RESV content (total concentration) in the colloidal suspensions was calculated from the drug concentration in the methanolic solutions and expressed as μg of compound/mL of suspension. The recoveries of CUR and RESV were expressed as the percentage of the total drug quantified in the suspensions relative to the initially added amount. Encapsulation efficiency (EE) was determined by subtracting the drug content detected in the supernatant—obtained after ultrafiltration/centrifugation using Ultrafree-MC membranes (100,000 NMWL; Millipore, Billerica, MA, USA)—from the total drug concentration measured in the colloidal dispersions. All analyses were performed in triplicate.

### 2.5. Stability Test

To monitor the stability of the selected NLC_cur+resv, the formulation was stored at room temperature (22–24 °C) over time (0, 30, and 90 days), during which physicochemical characteristics such as particle size, zeta potential, polydispersity index, quantification of bioactive compound content, and pH were evaluated. In addition, an accelerated stability test under mechanical stress was performed during these periods to assess physicochemical characteristics and detect possible macroscopic phase separation. In this study, the samples were centrifuged at 6000 rpm for 30 min [42].

### 2.6. In Vitro Release of CUR and RESV from NLC

For the release assay, the dialysis membrane method was employed [43]. Briefly, 2 mL of the NLC_cur+resv formulation was placed into dialysis bags (MWCO 10,000 Da, Sigma-Aldrich^®^, USA) with the ends securely closed. The release medium was selected to simulate the conditions of the NLC delivery site: a potassium phosphate buffer at pH 5.5 (approximating healthy skin pH, which ranges from 4.7 to 5.7) and polyethylene glycol 400 (PEG400) as a co-solvent to enhance the solubility of the compounds (sink conditions), mixed at a ratio of 70:30 (*v*/*v*) [44].

The dialysis bags were immersed in 170 mL of release medium, maintained under magnetic stirring at 600 rpm and 34 ± 2 °C for 72 h. Samples (1 mL) were withdrawn at predetermined time points (1, 3, 6, 12, 24, 48, and 72 h) and immediately replaced with an equal volume of fresh medium. Subsequently, 100 µL of mobile phase was added to each sample to ensure chromatographic compatibility, adjust the pH, and prevent degradation before injection, the samples were then analyzed by HPLC. The experiment was carried out in triplicate. The amounts of CUR and RESV released were expressed as percentages and plotted as a function of time (h). The release data were then fitted to zero-order, first-order, and Higuchi kinetic models using the following equations:

(2) DQ=Q0+Kt(Zero−order model)

(3) lnQ=lnQ0−Kt(First−order model)

(4) $$Q=\frac{K1}{2t}(Higuchi\;model)$$

In this model, Q represents the quantity of drug released over time t, while Q_0_ refers to the initial amount of the bioactive compound. The parameter K is the release rate constant specific to the model. Additionally, the residual sum of squares (RSS) is used to assess the goodness of fit of the mathematical model to the experimental data. When comparing models on the same scale, a lower RSS value suggests a better fit, indicating that the model more accurately represents the observed data within a linear regression context.

### 2.7. Ex Vivo Permeation and Retention of CUR and RESV in Intact and Impaired Porcine Skin

The permeation and retention experiments were conducted using Franz diffusion cells [38]. Porcine ear skin served as the membrane. Ears were sourced from a local slaughterhouse in Pelotas, RS. After removing the subcutaneous tissue and hair with scissors and a scalpel, the ears were trimmed into circular sections. The experiment was conducted under controlled conditions at 32 ± 2.0 °C, with continuous agitation at 500 rpm for 12 h, in accordance with OECD guideline 428 [45]. Before mounting, the skin samples were hydrated with pH 7.4 PBS for 15 min at room temperature. The circular porcine skin disks were then positioned in the Franz diffusion cells between the donor and receptor compartments, covering an effective area of 2.268 cm^2^.

The receptor chamber was filled with a potassium phosphate buffer at pH 5.5 and a PEG-400 mixture (70:30 *v*/*v*) to maintain sink conditions. In the donor compartment, 350 μL of CLN_cur+resv or cur+resv formulations (equivalent concentrations to the CLN) mixed in water and 0.1% xanthan gum was applied. After 12 h, a portion of the receptor fluid was collected, and the skin was removed from the cell. To assess the retention of CUR and RESV within the tissue, the treated area of the porcine skin was finely chopped with a scalpel and transferred to a volumetric flask containing methanol. The sample was sonicated for 30 min and subsequently stirred overnight [46]. Since dermatoses can affect different layers of the skin, experiments were also performed on impaired tissues to determine the total extent of permeation/retention of CUR and RESV under these conditions. Previous studies have established the use of adhesive tape stripping to remove the stratum corneum and simulate skin impairment [47]. For the impaired skin evaluation, the stratum corneum was removed using adhesive tape, employing 20 strips (Scotch® brand, 3M) before the experiment. All analyses were conducted in triplicate. Finally, the receptor fluid and skin extraction samples were passed through a 0.45 µm membrane filter (Millipore Corporation, Billerica, MA, USA) and subsequently quantified by HPLC.

### 2.8. In Vitro Investigation of the Antioxidant Activity of CUR and RESV Incorporated into NLCs

#### 2.8.1. DPPH Radical Scavenging Capacity

The DPPH (2,2-diphenyl-1-picrylhydrazyl) radical scavenging capacity was assessed following a modified procedure adapted from Kim et al. (2024) [48]. A 100 µM DPPH solution in methanol was prepared. The NLC formulations, as well as free CUR and RESV solubilized in methanol, were diluted (1:10; 1:20; 1:40 and 1:80) to obtain final concentrations of 10, 20, 40 and 80 µg/mL of each active compound. For the assay, 100 µL of each diluted sample was combined with 900 µL of DPPH solution and kept in the dark at room temperature for 60 min. Absorbance was then recorded at 517 nm using a UV–Vis spectrophotometer (Lambda 25, Perkin Elmer Inc., USA). The percentage of DPPH radical-scavenging capacity was calculated according to Equation (5):

(5) $$DPPH\;innibition\left(\%\right)=\frac{\left(Abscontrol-Abssample\right)}{Abscontrol}\times 100$$

where Abs_control_ is the mixture of methanol and DPPH solution, and Abs_sample_ is the mixture of NLC_cur+resv and DPPH solution.

Methanol was used as the negative control (vehicle blank), and an NLC_blank formulation (without CUR and RESV) was evaluated at matching excipient concentrations (10–80 µg/mL) to ensure no antioxidant interference from the formulation components. These controls were run in triplicate, showing minimal inhibition (<5%) to affirm the method’s reliability.

Trolox was used as the positive control (standard antioxidant reference) at concentrations of 10, 20, 40, and 80 µg/mL, following the identical procedure to construct a calibration curve for Trolox Equivalent Antioxidant Capacity (TEAC). The DPPH assay for Trolox was performed in triplicate, allowing direct comparison of the NLC formulations’ scavenging efficiency.

#### 2.8.2. ABTS Radical Scavenging Capacity

The ABTS (2,2′-Azino-bis(3-ethylbenzothiazoline-6-sulfonic acid)) radical-scavenging activity was determined according to the method described by Hussen et al. (2023) [49], with slight adjustments. The ABTS radical cation (ABTS•^+^) was produced by combining a 7 mM ABTS solution with 2.45 mM potassium persulfate and allowing the reaction to proceed in the dark at room temperature for 12–16 h. The resulting ABTS•^+^ solution was then diluted in ethanol until reaching an absorbance of 0.70 ± 0.02 at 734 nm, measured on a UV–Vis spectrophotometer (Lambda 25, Perkin Elmer Inc., USA). The NLC formulations, as well as free CUR and RESV solubilized in methanol, were prepared at the same concentrations used for the DPPH assay (10–80 µg/mL of each active compound). For the assay, 50 µL of each diluted sample was mixed with 950 µL of ABTS•^+^ solution and allowed to react at room temperature for 30 min. Absorbance was then measured at 734 nm. The percentage reduction in the ABTS radical was determined as described in Equation (6):

(6) $$ABTS\;innibition\left(\%\right)=\frac{\left(Abscontrol-Abssample\right)}{Abscontrol}\times 100$$

where Abs_control_ is the mixture of ethanol and ABTS solution, and Abs_sample_ is the mixture of NLC_cur+resv and ABTS solution.

Ethanol served as the negative control (vehicle blank). At the same time, an NLC_blank formulation was tested at equivalent concentrations (10–80 µg/mL excipients) to confirm the absence of intrinsic antioxidant activity from the lipid matrix. Both controls were assayed in triplicate and showed negligible inhibition (<5%) to validate assay specificity.

Trolox was employed as the positive control at concentrations of 10, 20, 40, and 80 µg/mL, using the same protocol to generate a standard curve for TEAC determination. The ABTS assay for Trolox was conducted in triplicate, enabling quantitative assessment of the NLC’s antioxidant potential relative to the standard.

### 2.9. In Vitro Evaluation of Cytotoxicity and Cell Proliferation Induced by CUR and RESV from NLC Using MTT and SRB Assays

#### 2.9.1. Cells and Culture Conditions

In vitro assays were conducted using an immortalized human keratinocyte line (HaCaT) supplied by the Experimental Oncology Laboratory of the Cancer Institute of São Paulo (ICESP; São Paulo, Brazil). The cells were cultured in high-glucose DMEM supplemented with 10% fetal bovine serum (FBS), 1% penicillin–streptomycin, and 1% amphotericin B. Cultures were maintained at 37 °C in an atmosphere of 5% CO_2_ and 95% relative humidity. Routine maintenance was performed twice weekly to prevent excessive cell confluence.

#### 2.9.2. Treatments and Experimental Groups

Cell viability and proliferation were assessed by measuring mitochondrial metabolism and cell density via protein content, respectively. For this, HaCat cells were seeded in 96-well plates (8 × 10^4^ cells/mL) with high-glucose DMEM medium, supplemented with 10% FBS and 1% antibiotic/antifungal solution. Cells were incubated at 37 °C in a 5% CO_2_ atmosphere with 95% humidity for 24 h to promote cell adhesion to the culture plates. After this period, the culture medium was replaced with fresh medium, and the cells were subsequently treated with NLC_blank or NLC_cur+resv at concentrations of 0.08, 0.4, 0.8, 4, and 8 µg/mL. Both formulations were prepared for treatment in FBS-free DMEM, and the control group was treated only with culture medium.

#### 2.9.3. Cell Viability Assay

The MTT assay (3-(4,5-Dimethyl-2-thiazolyl)-2,5-diphenyl-2H-tetrazolium bromide) was performed 24 h after treatments to assess cell viability through mitochondrial metabolism [50]. After the exposure period, the cells were washed twice with phosphate-buffered saline (PBS) and incubated with a solution containing 5 mg/mL MTT for 3 h. After the incubation period, the MTT solution was discarded, and 150 µL of dimethyl sulfoxide (DMSO) was added to each well to dissolve the formazan crystals. The absorbance was subsequently read at 490 nm using a microplate reader (BioTek Instruments, Winooski, VT, USA).

#### 2.9.4. Cell Proliferation Assay

Cell proliferation was evaluated using the sulforhodamine B (SRB) assay, which quantifies cellular protein content and provides a quantitative measure of cell density [51]. Thus, after 24 h of exposure, the cells were washed twice with PBS and fixed in 4% (*w*/*v*) formalin for 10 min. Subsequently, the cells were rewashed with PBS and incubated for 1 h in a solution of 1% acetic acid containing 0.4% SRB (*w*/*v*). Subsequently, the wells were rinsed with deionized water to remove any unbound dye. The plates were then allowed to dry at room temperature, and the protein-bound SRB was solubilized using 1% SDS (sodium dodecyl sulfate). Absorbance was measured using a fluorimeter at 540 nm (Filter Max F5, Molecular Devices, Molecular Devices, LLC; San Jose, CA, USA).

## 3. Statistical Analyses

Statistical analysis of the factorial design was performed using Python 3.12 (Python Software Foundation) in Google Colab (Google Research), employing the Pandas 2.2.2, NumPy 2.0.2, and StatsModels libraries 0.14.6. Multiple linear regression (OLS—Ordinary Least Squares) was used to estimate coefficients, standard errors, and *p*-values for each main effect and interaction, with *p* < 0.05 considered statistically significant. The main and interaction effects of the independent variables on the dependent responses were further evaluated by analysis of variance (ANOVA). Model adequacy was assessed using the coefficient of determination (R^2^), adjusted R^2^, and lack-of-fit tests. Responses were normalized to enable comparison across scales, and a total normalized score was calculated to identify the optimal overall experimental design. Radar plots were generated to visualize all experiments simultaneously, identify the best outcome for each response, and highlight the optimal overall experiment, facilitating interpretation of the combined effects of the factors and selection of optimal conditions.

For stability tests and all bioactive compound release experiments, measurements were performed in triplicate, and the data are expressed as mean ± standard deviation (SD). Statistical analyses were carried out using GraphPad Prism 8.0.2 (GraphPad Software, San Diego, CA, USA). For comparisons among multiple groups, one-way analysis of variance (ANOVA) followed by Tukey’s post hoc test was applied. A *p*-value < 0.05 was considered statistically significant.

## 4. Results

### 4.1. Development, Characterization and Factorial Planning

**Figure 1:**
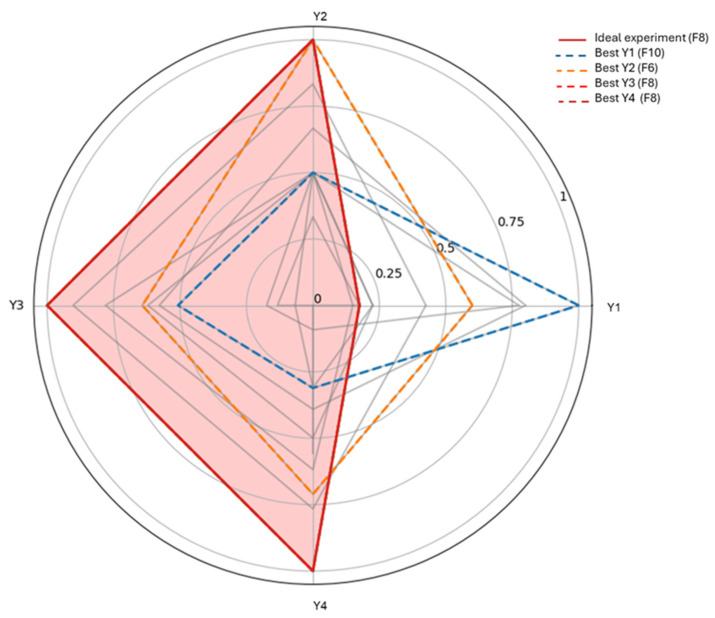
**Radar plot of experiments with normalized responses (Y1–Y4), where Y1 = particle size, Y2 = polydispersity index, Y3 = curcumin recovery (%), and Y4 = resveratrol recovery (%).** Each axis represents a dependent variable normalized to the [0, 1] range. Values closer to 1 indicate better performance for that response. All experiments are shown in gray, while the overall optimal experiment (with the highest sum of normalized responses) is highlighted in red. Dashed lines indicate the best individual experiment for each response, facilitating visualization of the optimal conditions for each variable.

**Figure 2:**
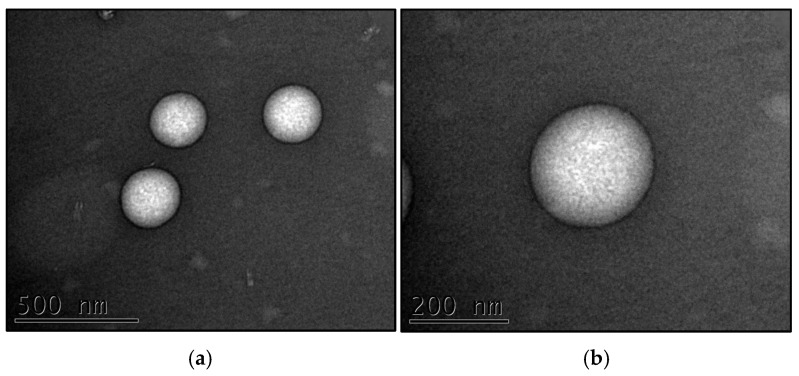

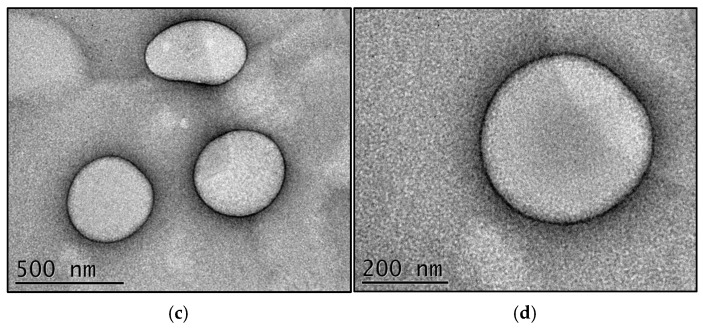
**Transmission electron micrographs of the NLC_cur+resv (a,b) and NLC_blank (c,d):** Images (**a**,**c**) were acquired at 50,000× magnification, while images (**b**,**d**) were acquired at 100,000× magnification. All micrographs were obtained 15 days after preparation of the formulations.

All proposed formulations were successfully developed by high-pressure homogenization. The drug content recovery capacity ranged from 53.20 to 86.20% for CUR and from 59.76 to 88.50% for RESV. Particle size varied slightly, between 269 and 303 nm, and the polydispersity index remained below 0.3 in all formulations (Table 3). The formulations were prepared according to a fractional factorial design. After obtaining the results, commands were generated for statistical analysis. The first commands consisted of (i) displaying each response; (ii) identifying the best experiment for each response; and (iii) performing a global analysis of the main effects, including two-factor interactions (The results of the round of analysis can be viewed at Table S3). Based on this analysis, the statistical results were evaluated; the main ones are presented in Figure 1, a radar plot of the experiments, and the morphology of the ideal formulation is visualized in Figure 2.

According to the study, multicollinearity was observed among the factors and interactions, as expected in fractional factorial designs, with factor X3 (castor oil) exerting the greatest influence on the formulations. X3 directly affected Y3 (CUR-loading capacity, *p* = 0.002), while X1, X2, X4, and their combinations had no significant effect; the model explained 97.4% of the variability (R^2^ = 0.974; adjusted R^2^ = 0.914). X3 also influenced Y4 (RESV-loading capacity, *p* = 0.008). X4 (shea butter) showed a trend toward an effect (*p* = 0.072), but it was not significant at the 0.05 level, and the other variables and interactions did not impact Y4. The model accounted for 95.2% of the variance (R^2^ = 0.952; adjusted R^2^ = 0.839), indicating that X3 predominantly determines Y4. Y1 (particle size) exhibited high residual variability, with no significant effect of the factors studied. Y2 (polydispersity index) was primarily influenced by X3, with contributions from X2, X4, and some interactions. Although the model suggested that higher polydispersity values would yield better responses, indices below 0.3 indicate monodisperse systems and greater stability, consistent with this limit [36].

#### Transmission Electron Microscopy (TEM)

The TEM micrographs obtained by negative staining revealed the size and morphology of NLC_cur+resv and NLC_blank, which were selected according to a factorial design. The nanocarrier was spherical, with dimensions and a polydispersity index in good agreement with DLS results.

### 4.2. Stability Study

**Figure 3:**
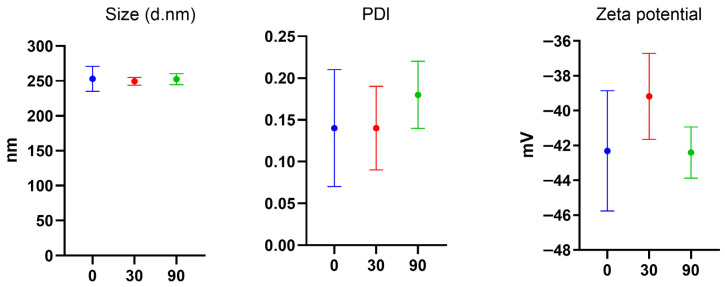
**Stability test of NLC_cur+resv over 90 days.** All measurements were performed in triplicate and are reported as mean ± SD; no significant differences were observed among the time points within the 95% confidence interval. (d. nm) = particle diameter in nanometers.

The NLC_cur+resv formulation was stored at room temperature (22–24 °C) and evaluated at 0, 30, and 90 days. The physicochemical characterization and stability results are summarized in Table 4 and in Figure 3. Particle size, PDI, zeta potential, and drug content were used as indicators of formulation stability over time.

The variation in asset quantification is also within expectations. According to the results presented in Table 4, the NLC_cur+resv formulation demonstrated good stability over 90 days of storage, showing no significant variations in physicochemical parameters. Furthermore, the mechanical stress test showed no evidence of phase separation, creaming, or sedimentation after centrifugation (6000 rpm, 30 min). Particle size and polydispersity index exhibited minimal variations (<10%), and the zeta potential remained within the same magnitude. The absence of macroscopic and physicochemical alterations indicates that the lipid nanocarrier dispersion is mechanically robust and physically stable under high-stress conditions.

### 4.3. In Vitro Bioactive Compounds Release

**Figure 4:**
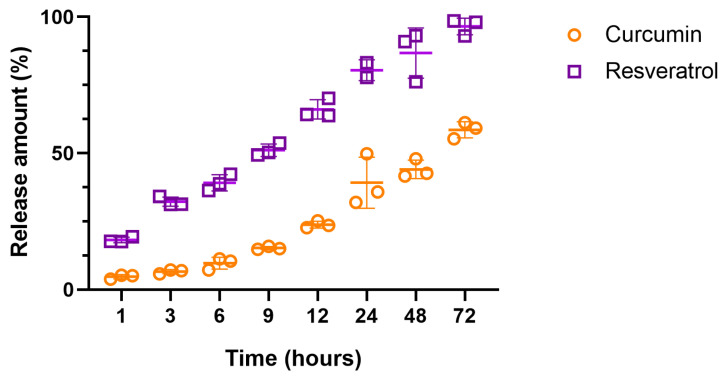
In vitro release profiles of the bioactive compounds (CUR and RESV), expressed as individual values, mean ± SD (n = 3).

The in vitro release of the compounds (CUR and RESV) from NLCs was evaluated at 34 ± 2 °C by dialysis in an appropriate receptor medium, as shown in Figure 4 and Table S4 (Supplementary Material). The release of both actives was detected as early as the first hour, with a continuous increase observed up to 72 h, indicating a sustained release profile. RESV exhibited a maximum release of 97 ± 3% at 72 h, suggesting complete release from the nanocarrier. Curcumin also exhibited a sustained-release pattern; however, its release was slower, reaching a maximum of 58.6 ± 2.9% at the same time point. The chosen receptor medium (buffered solution, pH 5.5, containing PEG 400) demonstrated the nanocarrier’s capability to release the actives in a controlled and sustained manner. The pH (5.5) and constant temperature (34 ± 2 °C) were selected to mimic physiological skin conditions, under which the formulations exhibited appropriate characteristics for dermatological delivery. In the analysis of CUR and RESV release kinetics from formulations, all time points were used to construct the graphs and perform linear regression.

**Figure 5:**
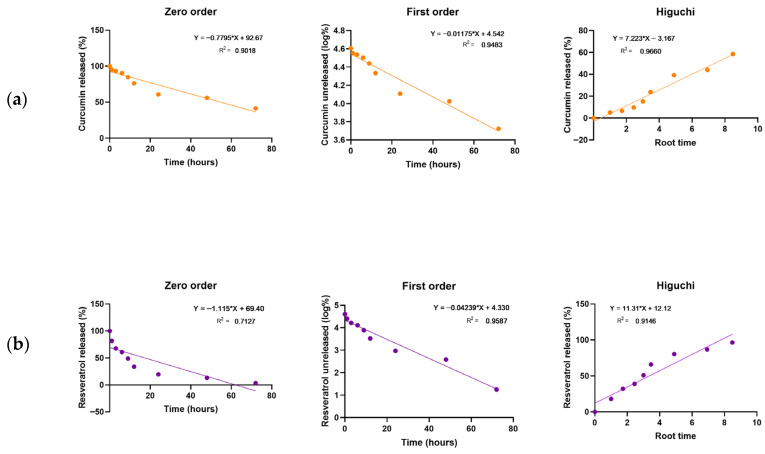
Release kinetics from NLC_cur+resv: (**a**) Curcumin and (**b**) Resveratrol. The corresponding kinetic curves, fitted equations, and R^2^ values (coefficient of determination) are presented for each model evaluated.

The in vitro release profiles of CUR and RESV from the NLC formulation were analyzed using the zero-order, first-order, and Higuchi kinetic models to elucidate the release mechanism. The higher R^2^ value indicates a better fit of the data (Figure 5) [38,52]. Among the evaluated models, the Higuchi model provided the best fit for CUR (R^2^ = 0.9660), indicating that CUR release is primarily governed by a diffusion-controlled mechanism within the lipid matrix (Table 5). In contrast, the first-order model exhibited the best correlation for RESV (R^2^ = 0.9587), suggesting that its release rate depends on the concentration of the active compound remaining in the carrier.

### 4.4. Ex Vivo Permeation and Retention Study in Intact and Impaired Skin

**Figure 6:**
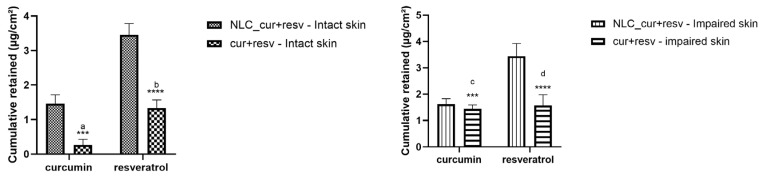
Mass of CUR and RESV retained in porcine skin ear after 12 h of permeation test in a Franz-type diffusion cell. The CUR and RESV were extracted with methanol overnight. Statistical analysis was performed using one-way ANOVA followed by Tukey’s multiple comparisons test. Significant differences are indicated as: *** *p* < 0.001, **** *p* < 0.0001. For CUR, significant comparisons included: NLC_cur+resv (intact) vs. cur+resv (intact) a *** *p* < 0.001 and cur+resv (intact) vs. cur+resv (impaired) c *** *p* < 0.001. For RESV, significant differences were found between: NLC_cur+resv (intact) vs. cur+resv (intact) b **** *p* < 0.0001 and NLC_cur+resv (impaired) vs. cur+resv (impaired) d **** *p* < 0.0001. NLC_cur+resv (nanoencapsulated) cur+resv (non-encapsulated).

At the end of the permeation experiment (12 h), neither CUR nor RESV was detected in the receptor fluid. After methanolic extraction of the compounds retained in the skin, the retention results are presented in Table 6 and Figure 6. The impaired skin condition was obtained by a tape-stripping technique prior to the permeation assay.

### 4.5. Antioxidant Activity

#### 4.5.1. DPPH

The antioxidant activity of NLC_cur+resv was evaluated based on its ability to scavenge the DPPH radical. Antioxidant compounds interact with DPPH via electron or hydrogen-atom transfer, reducing it to 1,1-diphenyl-2-picrylhydrazine. This reaction leads to a color change from deep purple to pale yellow, indicating the active compound’s capacity to neutralize free radicals [53].

**Figure 7:**
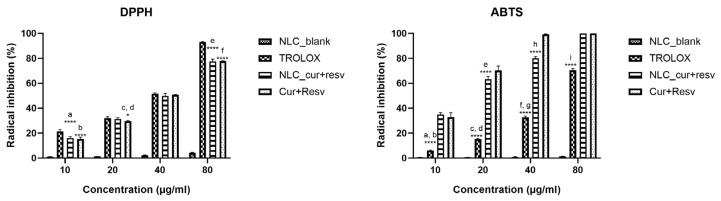
Percentage inhibition of DPPH and ABTS radicals by NLC_blank, NLC_CUR+RESV, CUR+RESV (non-encapsulated), and Trolox, expressed as mean ± SD (n = 3). Statistical analysis was performed using two-way ANOVA followed by Tukey’s multiple comparison test. Significant differences are indicated as: * *p* < 0.05 and **** *p* < 0.0001. DPPH assay: a: *p* < 0.0001, Trolox vs. NLC_CUR+RESV; b: *p* < 0.0001, Trolox vs. CUR+RESV; c: *p* < 0.05, Trolox vs. CUR+RESV; d: *p* < 0.05, NLC_CUR+RESV vs. CUR+RESV; e: *p* < 0.0001, Trolox vs. NLC_CUR+RESV; f: *p* < 0.0001, Trolox vs. CUR+RESV. ABTS assay: a: *p* < 0.0001, NLC_CUR+RESV vs. Trolox; b: *p* < 0.0001, CUR+RESV vs. Trolox; c: *p* < 0.0001, NLC_CUR+RESV vs. Trolox; d: *p* < 0.0001, CUR+RESV vs. Trolox; e: *p* < 0.0001, CUR+RESV vs. NLC_CUR+RESV; f: *p* < 0.0001, NLC_CUR+RESV vs. Trolox; g: *p* < 0.0001, CUR+RESV vs. Trolox; h: *p* < 0.0001, CUR+RESV vs. NLC_CUR+RESV; i: *p* < 0.0001, NLC_CUR+RESV vs. Trolox.

The formulation exhibited an apparent concentration-dependent antioxidant effect, with inhibition values of 16.14%, 31.46%, 49.94%, and 77.54% at concentrations of 10, 20, 40, and 80 µg/mL, respectively (Figure 7). The results demonstrated a strong linear correlation between concentration and scavenging effect (y = 0.8538x + 12.128; R^2^ = 0.9779). The calculated IC_50_ value of 44.88 µg/mL (total CUR + RESV) indicates a high antioxidant potential of the NLC_cur+resv. The NLC_blank had no significant antioxidant capacity. Free CUR and RESV solubilized in methanol also exhibited concentration-dependent DPPH radical scavenging activity, with inhibition values of 15.35 ± 1.41%, 29.57 ± 0.52%, 50.81 ± 0.20%, and 77.67 ± 0.42% at 10, 20, 40, and 80 µg/mL, respectively. The corresponding linear regression equation was y = 0.8656x + 10.893 (R^2^ = 0.9757). The calculated IC_50_ value of 45.17 µg/mL (total CUR + RESV) indicates a comparable antioxidant profile between the free and nanoencapsulated forms.

As a reference standard, Trolox exhibited a dose-dependent scavenging profile, with inhibition values of 21.49%, 32.06%, 51.71%, and 93.14% at 10, 20, 40, and 80 µg/mL, respectively (Figure 7). The linear regression equation was y = 1.0211x + 11.308 (R^2^ = 0.999), yielding an IC_50_ of approximately 37.89 µg/mL. When compared to Trolox, the lower IC_50_ obtained for NLC_cur+resv (44.88 µg/mL) confirms that the nanoencapsulated compounds retain a substantial radical-scavenging capacity in the DPPH system. Overall, these findings demonstrate that nanoencapsulation effectively preserves the antioxidant potential of CUR and RESV. This test showed that nanoencapsulation of CUR and RESV effectively preserved and enhanced the radical-scavenging properties of the active compounds in the DPPH assay.

#### 4.5.2. ABTS

The ABTS radical cation is generated through the oxidation of ABTS by potassium persulfate. In the presence of antioxidants capable of donating hydrogen atoms, the radical cation is reduced. This reaction decolorizes the blue ABTS radical, reflecting the free-radical scavenging activity of the tested compounds [54].

The formulation demonstrated a strong and concentration-dependent scavenging effect, with inhibition values of 34.99%, 63.30%, 80.08%, and 100% at concentrations of 10, 20, 40, and 80 µg/mL, respectively (Figure 7). A good linear correlation was observed between the concentration and percentage inhibition (y = 0.8279x + 38.549, R^2^ = 0.8675). The calculated IC_50_ value of 13.83 µg/mL (total CUR + RESV) indicates a markedly higher antioxidant potential when compared to the DPPH assay, suggesting that NLC_cur+resv exhibits a more efficient electron or hydrogen donating capacity against the ABTS radical. Free CUR and RESV solubilized in methanol also exhibited pronounced ABTS radical scavenging activity, with inhibition values of 32.96%, 71.20%, and 99.30% at concentrations of 10, 20, and 40 µg/mL, respectively. Because inhibition at 40 µg/mL nearly achieved complete radical scavenging, the 80 µg/mL concentration was excluded from the linear regression analysis and IC_50_ calculation to avoid saturation effects. The corresponding linear regression equation was y = 2.0962x + 18.909 (R^2^ = 0.9245), resulting in an IC_50_ value of 14.83 µg/mL (total CUR + RESV). These results indicate a comparable antioxidant capacity between the free and nanoencapsulated forms in the ABTS assay. It was also observed that NLC_blank exhibited no significant scavenging activity, confirming that the observed effect was attributable exclusively to the active compounds.

As the reference standard, Trolox showed a dose-dependent response with inhibition values of 6.11%, 15.19%, 32.71%, and 70.45% at 10, 20, 40, and 80 µg/mL, respectively (Figure 7). The linear regression model was y = 0.919x − 3.3465 (R^2^ = 0.9997), yielding an IC_50_ of approximately 58.1 µg/mL. The NLC_cur+resv formulation exhibited a strong, concentration-dependent antioxidant effect, with an IC_50_ of 13.83 µg/mL, which is substantially lower than that of Trolox (58.1 µg/mL). This indicates a markedly higher ABTS-scavenging capacity for NLC_cur+resv. No significant inhibition was observed for the NLC_blank, confirming that the antioxidant activity originated exclusively from the active compounds.

### 4.6. Cytotoxicity and Cell Proliferation Results (MTT and SRB Assays)

**Figure 8:**
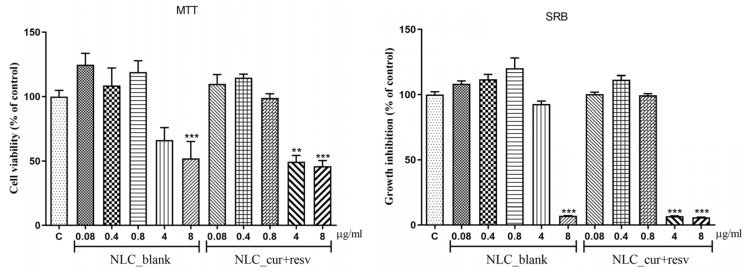
Analysis of cell viability and proliferation. MTT and SRB assays in the HaCat cell line 24 h after treatments with 0.08, 0.4, 0.8, 4, and 8 µg/mL of NLC_blank and NLC_cur+resv (n = 6) wells, three independent experiments. Data are expressed as mean ± S.E.M (Standard Error of the Mean). One-way ANOVA followed by Tukey–Kramer post hoc test for multiple comparisons. ** *p* < 0.01 and *** *p* < 0.001; differences from the control group (C). Bar patterns correspond to the different concentrations evaluated for each formulation (0.08, 0.4, 0.8, 4, and 8 µg/mL).

Cell viability and proliferation were evaluated in HaCat human keratinocytes after 24 h of exposure to 0.08, 0.4, 0.8, 4, and 8 µg/mL of NLC_blank or NLC_cur+resv. As shown in Figure 8, both MTT and SRB assays demonstrated reduced cell viability and proliferation at 8 µg/mL for NLC_blank, and at 4 and 8 µg/mL for NLC_cur+resv. At lower concentrations (up to 4 µg/mL for NLC_blank and up to 0.8 µg/mL for NLC_cur+resv), no reductions in metabolic activity or cell proliferation were observed. MTT and SRB results showed consistent response profiles across all tested groups.

## 5. Discussion

As stated in the Introduction, this study aimed to develop and optimize an NLC lipid carrier co-encapsulating CUR and RESV using a fractional factorial design, aiming to obtain a stable topical formulation with enhanced antioxidant and anti-inflammatory properties. Consistent with this objective, the results show that the multivariate approach efficiently identified an optimized formulation with nanometric particle size (~300 nm), low polydispersity (PDI < 0.3), high bioactive loading, and good physicochemical stability. The formulation also exhibited sustained release behavior, preserved antioxidant activity, enhanced retention of both compounds in intact and impaired skin, and acceptable cellular compatibility. Importantly, beyond selecting an optimal composition, the factorial design provided analytical insight into how excipient ratios governed critical formulation attributes, reinforcing the value of statistical optimization in rational nanocarrier development.

The size of nanocarriers influences their ability to penetrate and release bioactive compounds into the different layers of the skin. Consistent with this, the particle size obtained in our formulation (~300 nm) aligns with preferential deposition within the epidermis, which supports the observed retention amounts in permeation studies and reinforces the formulation’s suitability for topical, not transdermal, delivery. Previous studies have shown that nanocarriers with an average diameter of around 300 nm mainly reach the upper layers of the epidermis, which is the layer of skin most directly and visually affected by oxidative stress diseases and skin inflammations [55,56].

Zeta potential is the electric potential at the interface between a particle and the dispersing medium and is an effective measure of the surface charge that contributes to colloidal stability. It reflects the strength of electrostatic repulsion between nearby particles and is crucial for predicting whether they will cluster. If repulsive electric double layers are insufficient to separate the particles, they can aggregate via van der Waals attraction. Generally, a zeta potential sufficient to stabilize a system is assumed to be around ± 30 mV [57,58]. The developed nanocarrier exhibited a zeta potential of −39.17 ± 2.47 mV, indicating strong electrostatic repulsion between particles, which contributes to the system’s colloidal stability and prevents aggregation. This high negative charge is also consistent with the stability observed over 90 days, reinforcing the role of the lipid-surfactant combination optimized in this study.

The pH of the formulation was 5–6, suitable for dermatological use, as the skin has a pH of 4.5–6; thus, the formulation avoids irritation, promotes hydration, and supports the recovery of injured skin [59,60]. Maintaining this physiological pH range is particularly relevant considering the intended continuous or repeated use of topical formulations.

The use of statistical design in formulation development not only reduced experimental workload and improved reproducibility but also increased the likelihood of achieving nanocarriers with optimal performance, including high drug content and a uniform particle-size distribution. More importantly, the multivariate analysis provided insight into how oils, butters, and surfactants influenced key physicochemical characteristics, allowing deeper interpretation beyond formulation development. The multivariate analysis applied in this study enabled the identification of key relationships between formulation variables, such as oils, butters, and surfactants, and the resulting physicochemical properties, underscoring how the strategic selection and combination of excipients directly influence the final characteristics of the nanocarrier [3,14,61].

In this formulation, the lipid excipients were selected for their dermal compatibility and ability to support the stability of the NLC system. Coconut oil, shea butter, and castor oil provide emollient and barrier-supporting fatty acids that help maintain the structural integrity of the lipid matrix. A small amount of vitamin E was added to enhance oxidative stability and protect the skin. Together, these components strengthen the formulation and complement the biological profiles of CUR and RESV [62,63,64,65,66].

An in vitro release assay was performed to characterize the profiles of CUR and RESV released from the NLC. Using the obtained data, the release profiles were analyzed, and the mathematical model that best describes the release was identified, considering zero-order, first-order, and Higuchi models. The results indicated that CUR followed a diffusion-controlled mechanism consistent with the Higuchi model (R^2^ = 0.9660; RSS = 113.01), suggesting a Fickian diffusion process through the solid lipid matrix, although the first-order model also showed a good correlation (R^2^ = 0.9483; RSS = 0.037), the higher coefficient of determination and mechanistic relevance support the Higuchi model as the most appropriate to describe the CUR release behavior. In contrast, RESV exhibited a concentration-dependent release profile best described by the first-order model (R^2^ = 0.9587; RSS = 0.381), indicating a non-Fickian diffusion mechanism. The distinction between the two release mechanisms suggests that each molecule interacts differently with the lipid matrix, likely due to differences in lipophilicity and molecular weight, which may influence their partitioning and mobility within the nanostructured system. This differential behavior may also affect their subsequent availability within the skin, and this aspect warrants further exploration in future in vitro and in vivo permeation and anti-inflammatory studies.

These findings demonstrate that the NLC system promotes a controlled and sustained release of both compounds, favoring prolonged bioavailability. Similar results were reported by Araujo et al. (2023) [38], who described the CUR release profile from NLC using a Higuchi model, corroborating our result. The study evaluated the difference in CUR release from a nanoemulsion and NLC. It showed that the presence of solid lipids in the nanocarrier matrix potentiates the prolonged release of CUR.

Additionally, Li et al. (2023) [67] investigated the release behavior of RESV from nanoliposomes under pH conditions comparable to those used in our study. They observed higher release percentages in slightly acidic media. Although liposomes and NLCs possess distinct structural organizations, this reference was considered relevant because it provides contextual information on how RESV behaves under similar pH conditions. In our formulation, the non-Fickian diffusion mechanism observed for RESV likely arises from a combination of its physicochemical properties and its distribution within the heterogeneous lipid matrix. As observed, RESV exhibited a faster release, while CUR displayed a more sustained release pattern, supporting the concept of an initial antioxidant effect followed by prolonged activity.

Furthermore, Palliyage et al. (2021) [68] developed solid lipid nanoparticles containing CUR and RESV with a mean diameter of 180.2 ± 7.7 nm via high-intensity shear homogenization, a technique distinct from our high-pressure homogenization for NLCs, and their stability was limited to two weeks, which is shorter than that of our formulation (90 days). Similarly, Coradini et al. (2014) [69] co-encapsulated the same bioactive compounds in polymeric nanocapsules with a lipid core, obtaining particles of approximately 200 nm, a drug load of 49 mg/mL/each, and a maximum release of 35% of CUR in 72 h. In their development, polymers were used to coat the oily core, whereas NLCs contain only a mixture of liquid and solid lipids. In comparison, the NLC_cur+resv formulation developed in this study exhibited high co-encapsulation capacity (>85 mg/mL for each active compound) and sustained, improved release profiles for both CUR and RESV, with approximately 59% and 97% released, respectively, after 72 h. These comparisons indicate that the systematic optimization employed in the present study was essential for achieving superior stability and release performance compared with previously developed nanocarriers containing CUR and RESV. A performance comparison with previously reported nanocarriers is summarized in Table S5 (Supplementary Material). Recent studies have further emphasized the importance of rational design and functional optimization of nanostructured delivery systems to achieve controlled and staged release, improved stability, and enhanced biological performance [70].

CUR and RESV are known for their multi-potential therapeutic applications [71,72]. This study evaluated the ability of these agents to neutralize free radicals, which play a significant role in the pathophysiology of various dermatoses. Oxidative stress plays a central role in the pathogenesis of multiple inflammatory skin disorders by inducing macromolecular damage and activating pro-inflammatory signaling pathways, such as NF-κB and MAPK, which enhance cytokine production (TNF-α, IL-1β, IL-6) and recruit inflammatory cells [22,28]. Skin conditions associated with oxidative stress include inflammatory diseases such as psoriasis, atopic dermatitis, and melasma, among others, as well as bacterial infections such as leprosy and fungal infections such as mycoses [73,74].

To validate the antioxidant activity of the NLC_cur+resv formulation, in vitro assays were performed using the DPPH and ABTS methods. Both tests demonstrated high free radical-scavenging capacity, highlighting the potential of co-encapsulation of CUR and RESV for neutralizing reactive oxygen species. The antioxidant assays confirmed that NLC_cur+resv preserved the radical-scavenging properties of the encapsulated compounds, whereas NLC_blank exhibited no significant activity, indicating that the effect is solely attributable to the actives. These findings corroborate previous reports that CUR nanoencapsulation does not impair, and may even enhance, its antioxidant activity [75].

When comparing the IC_50_ values of the nanoformulation to those of the free compounds, the results indicate that nanoencapsulation maintained the intrinsic activity of CUR and RESV. For DPPH, NLC_cur+resv exhibited an IC_50_ of 44.88 µg/mL (total cur + resv), which closely matched the value obtained for the free mixture (45.17 µg/mL). In ABTS, NLC_cur+resv presented an IC_50_ of 13.83 µg/mL, slightly lower than that of the free compounds (14.83 µg/mL), suggesting equal or marginally improved activity. Additionally, the nanoformulation outperformed Trolox in the ABTS assay (13.83 µg/mL vs. 58.1 µg/mL) but showed slightly lower potency in the DPPH assay (44.88 µg/mL vs. 37.89 µg/mL).

These differences align with the distinct reaction mechanisms and sensitivities of the assays. ABTS generally reacts more rapidly and completely with phenolic antioxidants, due to its compatibility with both hydrophilic and lipophilic molecules. At the same time, DPPH is more restricted and slower in its interaction kinetics [71,76,77]. The preserved and in some cases slightly improved antioxidant performance supports the premise that nanoencapsulation sustains the functional efficacy of CUR and RESV, potentially enhancing their activity through increased solubility and stability.

Shi et al. (2021) [78] observed an IC_50_ value of 16.97 mg/mL in zein nanocapsules containing RESV, reporting superior antioxidant activity compared to free RESV in the ABTS assay. The difference in IC_50_ concentration (IC_50_ = 13.83 µg/mL of each active compound) observed in our study may be related not only to the way RESV was nanoencapsulated but also to the synergistic action of CUR. Other studies suggest that modifying CUR solubility improves its antioxidant effects; for example, Kang et al. (2024) [79] investigated the antioxidant activities of free CUR and CUR associated with polyvinyl alcohol (PVA) using the DPPH and ABTS assays. The authors highlighted that CUR’s radical-scavenging activity was significantly enhanced in the presence of PVA, with this effect being more pronounced in aqueous systems, such as the ABTS assay, than in organic solvent-based systems, such as the DPPH assay. This observation reinforces the formulation’s improved performance in the ABTS system, supporting the interpretation that the assay’s hydrophilic environment favors the enhanced antioxidant response of the co-encapsulated compounds.

The permeation and retention results showed that both CUR and RESV remained confined to the skin, with no permeation into the receptor phase. The NLC_cur+resv formulation consistently yielded higher retention of both compounds than the non-encapsulated mixture, indicating that the nanocarrier enhances cutaneous deposition. Barrier disruption increased retention of the free mix, particularly for CUR, but did not significantly affect retention when the compounds were nanoencapsulated. These findings indicate that the NLC system promotes more efficient and controlled skin deposition and maintains a stable retention profile even under partial impairment of the stratum corneum [80,81].

The cytotoxicity and proliferation assays provided additional insights into the biological safety of the developed NLC system. A concentration-dependent response was observed in HaCat keratinocytes, with reductions in metabolic activity and proliferation occurring only at higher concentrations of NLC_blank (8 µg/mL) and NLC_cur+resv (4 and 8 µg/mL). These findings are consistent with reports showing that elevated lipid or surfactant loads in nanocarriers may induce cellular stress [82]. Therefore, our results indicate that treatments with up to 4 µg/mL of NLC_blank and up to 0.8 µg/mL of NLC_cur+resv do not present cytotoxic and cytostatic effects on the HaCat human keratinocyte cell line.

Although the present study successfully developed and optimized an NLC co-encapsulating CUR and RESV, certain limitations should be acknowledged. The investigation was primarily focused on physicochemical characterization and in vitro antioxidant assessment. Therefore, additional studies are warranted to evaluate in vivo performance, long-term stability under different storage and environmental conditions, and the formulation’s therapeutic efficacy in relevant skin models. Moreover, exploring the anti-inflammatory and regenerative effects using more physiologically relevant models would provide deeper insight into the system’s therapeutic potential. Future research will aim to extend the biological evaluation by exploring the system’s anti-inflammatory and regenerative potential, as well as its integration into topical delivery platforms for dermocosmetic and pharmacological applications. Despite these limitations, the findings clearly demonstrate that the rational design and statistical optimization of lipid-based nanocarriers constitute an effective strategy to improve the stability, bioactivity, and multifunctional performance of natural bioactive compounds.

## 6. Conclusions

This study successfully developed and optimized an NLC co-encapsulating CUR and RESV using a fractional factorial design approach. The application of experimental design enabled the identification of critical formulation variables, reducing experimental complexity while ensuring reproducibility in a multicomponent lipid system. The optimized formulation exhibited suitable physicochemical characteristics, including homogeneous nanometric particle size, low polydispersity, high bioactive loading, and good physicochemical stability during storage. The nanocarrier promoted sustained release of both compounds, with distinct release profiles reflecting their interactions within the lipid matrix. Skin permeation studies demonstrated enhanced retention of CUR and RESV within the skin, with no detectable permeation into the receptor phase, supporting the system’s suitability for topical delivery. In addition, the formulation preserved the antioxidant activity of the encapsulated compounds and showed cellular compatibility in human keratinocytes at formulation-relevant concentrations. Overall, these findings demonstrate that integrating experimental design strategies with lipid-based nanotechnology provides a robust and reproducible framework for developing topical nanocarriers co-loading natural antioxidants, thereby supporting further formulation-oriented and biological investigations under controlled experimental conditions.

## Figures and Tables

**Table 1:** Design layout of all formulations by 2^(4−1)^ fractional factorial design.

Sample ID	X1 * Sucrose Distearate	X2 Coconut Oil	X3 Castor Oil	X4 Shea Butter
F1	−1	−1	−1	−1
F2	1	−1	−1	1
F3	−1	1	−1	1
F4	1	1	−1	−1
F5	−1	−1	1	1
F6	1	−1	1	−1
F7	−1	1	1	−1
F8	1	1	1	1
F9	0	0	0	0
F10	0	0	0	0
F11	0	0	0	0

* X1–X4 correspond to the adjustable components listed in the table.

**Table 2:** Compositions of nanocarriers.

		Factorial Level		
		−1	0	1
Compounds		%	%	%
Oil phase	Sucrose distearate	2.5	3	3.5
	coconut oil	6.65	8.35	10
	castor oil	5.35	6.65	8
	shea butter	4	5	6
	sorbitan monooleate	1	1	1
	vitamin E	0.1	0.1	0.1
	curcumin	0.1	0.1	0.1
	resveratrol	0.1	0.1	0.1
Aqueous phase	heptyl glucoside (*w*/*v*)	2	2	2
	water purified *qs* *	100	100	100

* *qs* = quantity sufficient.

**Table 3:** Physicochemical characterization.

	Y1 Particle Size (d. nm) *	Y2 Polydispersity Index	Y3 Curcumin Recovery (%)	Y4 Resveratrol Recovery (%)
F1	294	0.17	59.20	62.40
F2	263	0.14	53.51	75.76
F3	272	0.17	55.69	68.67
F4	270	0.16	57.87	59.76
F5	269	0.17	79.03	77.52
F6	287	0.21	74.50	80.18
F7	280	0.19	83.01	81.76
F8	270	0.20	86.20	88.50
F9	295	0.18	72.40	71.00
F10	303	0.17	70.10	68.70
F11	272	0.17	73.81	74.10

* (d. nm) = particle diameter in nanometers.

**Table 4:** Physicochemical characterization and stability of NLC_cur+resv over 90 days.

Time (Days)	CUR Content (μg/mL)	CUR Recovery (%)	CUR EE (%)	RESV Content (μg/mL)	RESV Recovery (%)	RESV EE (%)	pH
**0**	886.74 ± 39.01	88.7 ± 3.9	99.79 ± 0.2	884.48 ± 25.27	88.4 ± 2.5	98.53 ± 0.4	5.84 ± 0.12
**30**	850.80 ± 29.36	85.8 ± 2.9	99.81 ± 0.2	865.45 ± 34.46	86.5 ± 3.4	97.88 ± 0.4	5.91 ± 0.2
**90**	855.52 ± 27.57	85.5 ± 2.75	99.22 ± 0.4	895.98 ± 20.09	89.5 ± 2.01	97.75 ± 0.4	5.99 ± 0.15

No statistical difference between the times; Values are expressed as mean ± SD (n = 3) CUR = curcumin; RESV = resveratrol; EE = encapsulation efficiency; SD = standard deviation; n = number of replicates.

**Table 5:** Kinetic parameters obtained from NLC_cur+resv release profiles.

	Curcumin		Resveratrol	
	R^2^	RSS	R^2^	RSS
Zero order	0.9018	326.22	0.7127	2470.16
First order	0.9483	0.037	0.9587	0.381
Higuchi	0.9660	113.01	0.9146	734.12
parameters	Higuchi		First order	
Equation	Y = 7.223X − 3.167		Y = −0.04239X + 4.330	
*K 72* h (h^−1^) *	0.01		0.04	

* The value of K was determined considering the Higuchi model for curcumin and the first order model for resveratrol. R^2^ = coefficient of determination; RSS = residual sum of squares; K = release rate constant.

**Table 6:** Ex vivo skin permeation and retention of CUR and RESV in intact and impaired (tape-stripped) skin after 12 h of permeation.

	Curcumin		Resveratrol	
	NLC	FREE	NLC	FREE
Intact skin retained (µg/cm^2^)	1.46 ± 0.25	0.26 ± 0.15 a	3.45 ± 0.33	1.33 ± 0.23 b
Impaired skin retained (µg/cm^2^)	1.62 ± 0.21	1.43 ± 0.15 c	3.44 ± 0.48	1.57 ± 0.40 d
Permeated (µg/cm^2^)	ND *	ND *	ND *	ND *

Results are presented as mean ± SD (n = 3). Statistical analysis is described in the Figure and indicated by different letters. NLC = nanostructured lipid carriers; FREE = non-encapsulated compound; * ND = not detected; SD = standard deviation; n = number of replicates.

## Data Availability

The original contributions presented in this study are included in the article/Supplementary Material. Further inquiries can be directed to the corresponding authors.

## Supplementary Materials

The following supporting information can be downloaded at: https://www.mdpi.com/article/10.3390/pharmaceutics18010109/s1; Table S1: Data related to the LOD. LOQ. Slope, and interception for curcumin and resveratrol chromatographic analysis method; Table S2: Full factorial design with responses; Table S3: Global Analysis: Main Effects + 2-Factor Interactions; Table S4: In vitro release profiles of the bioactive compounds (CUR and RESV), expressed as mean ± SD (n = 3), and Table S5: Comparative analysis of nanocarriers co-encapsulating CUR and RESV.
