# Supplementary material for: Design and Factorial Optimization of Curcumin and Resveratrol Co-Loaded Lipid Nanocarriers for Topical Delivery
Source: Pharmaceutics. 2026 Jan 15;18(1):109. doi: 10.3390/pharmaceutics18010109 (PMC12845360; doi:10.3390/pharmaceutics18010109)
Supplement: Supplementary file 1 [file pharmaceutics-18-00109-s001.zip › pharmaceutics-3988774-supplementary.pdf]

Supplementary Materials: Table S1: Data related to the LOD. LOQ. slope. and interception for curcumin and resveratrol chromatographic analysis method; Table S2: Full factorial design with responses; Table S3: Global Analysis: Main Effects + 2-Factor Interactions; Table S4: *In vitro* release profiles of the bioactive compounds (CUR and RESV), expressed as mean ± standard deviation (n=3); **Table S5: Comparative analysis of nanocarriers co-encapsulating CUR and RESV.**

Table S1: Data related to the LOD. LOQ. slope. and interception for curcumin and resveratrol chromatographic analysis method.

	**Parameters**	**Validation Results**
	Linearity	Calibration range (µg/ml): 0.25–10.0
		Y= 44564x + 2729 R^2: 0.9959
**Curcumin**	LOD	0.05 µg/ml
	LOQ	0.15 µg/ml
	SLOP	44564 ± 280.09
	P > 0.5	
	Intercept	2729 ± 650.18
	P > 0.5	
	Time retention	6.90 Min
	Linearity	Calibration range (µg/ml): 0.25–10.0
		Y= 6681X + 36069 R^2= 0.9982
**Resveratrol**	LOD	0.08 µg/ml
	LOQ	0.23 µg/ml
	SLOP	6681 ± 3088.58
	P > 0.5	
	Intercept	36069 ± 1590.64
	P > 0.5	
	Time retention	2.11 min

Table S2: Full factorial design with responses

						x1	x2	x3	x4				Y1	Y2	Y3	Y4
						SS SP30	Coconut Oil	Castor Oil	Shea Butter	Spam + Curcumin + Resveratrol	Sum of x1 - x4	water qs 100%	particle size (d.nm)	polydispersity index	Drug recovery cur (%)	Drug recovery resv (%)
	assays	x1	x2	x3	x4	%	%	%	%	%	%	%	nm		%	%
**F1**	1	-1	-1	-1	-1	2,5	6,7	5,3	4	1,2	19,7	80,3	294	0,17	59,20	62,40
**F2**	2	1	-1	-1	1	3,5	6,7	5,3	6	1,2	22,7	77,3	263	0,14	53,51	75,76
**F3**	3	-1	1	-1	1	2,5	10	5,3	6	1,2	25,0	75,0	272	0,17	55,69	68,67
**F4**	4	1	1	-1	-1	3,5	10	5,3	4	1,2	24,0	76,0	270	0,16	57,87	59,76
**F5**	5	-1	-1	1	1	2,5	6,7	8	6	1,2	24,4	75,6	269	0,17	79,03	77,52
**F6**	6	1	-1	1	-1	3,5	6,7	8	4	1,2	23,4	76,6	287	0,2	74,50	80,18
**F7**	7	-1	1	1	-1	2,5	10	8	4	1,2	25,7	74,3	280	0,19	83.01	81,76
**F8**	8	1	1	1	1	3,5	10	8	6	1,2	28,7	71,3	270	0,2	86,20	88,50
**F9**	9	0	0	0	0	3	8,35	6,65	5	1,2	24,2	75,8	295	0,18	72,40	71,00
**F10**	10	0	0	0	0	3	8,35	6,65	5	1,2	24,2	75,8	303	0,17	70,10	68,70
**F11**	11	0	0	0	0	3	8,35	6,65	5	1,2	24,2	75,8	272	0,17	73,81	74,10

Table S3: Global Analysis: Main Effects + 2-Factor Interactions

--- Analyzing Y1 ---

Model Summary (Coefficients and p-values): OLS Regression Results

==

Dep. Variable:	Y1	R-squared:	0.441
Model:	OLS	Adj. R-squared:	-0.862
Method:	Least Squares	F-statistic:	0.3388
Date:	Tue, 21 Oct 2025	Prob (F-statistic):	0.892
Time:	14:53:54	Log-Likelihood:	-40.239
No. Observations:	11	AIC:	96.48
Df Residuals:	3	BIC:	99.66
Df Model:	7		
Covariance Type:	nonrobust		

==

	coef	std err	t	P>|t|	[0.025	0.975]
Intercept	279.5455	5.418	51.592	0.000	262.302	296.789
x1	-3.1250	6.354	-0.492	0.657	-23.345	17.095
x2	-2.6250	6.354	-0.413	0.707	-22.845	17.595
x3	0.8750	6.354	0.138	0.899	-19.345	21.095
x4	-7.1250	6.354	-1.121	0.344	-27.345	13.095
x1:x2	0.0625	3.177	0.020	0.986	-10.048	10.173
x1:x3	2.5625	3.177	0.807	0.479	-7.548	12.673
x1:x4	0.5625	3.177	0.177	0.871	-9.548	10.673
x2:x3	0.5625	3.177	0.177	0.871	-9.548	10.673
x2:x4	2.5625	3.177	0.807	0.479	-7.548	12.673
x3:x4	0.0625	3.177	0.020	0.986	-10.048	10.173

==

Omnibus:	11.624	Durbin-Watson:	1.445
Prob(Omnibus):	0.003	Jarque-Bera (JB):	6.199

Skew: 1.725 Prob(JB): 0.0451

Kurtosis: 4.275 Cond. No. 9.85e+16

==

Notes:

[1] Standard Errors assume that the covariance matrix of the errors is correctly specified.

[2] The smallest eigenvalue is 1.65e-33. This might indicate that there are

strong multicollinearity problems or that the design matrix is singular.

ANOVA:

	df	sum_sq	mean_sq	F	PR(>F)
x1	1.0	78.125000	78.125000	0.241910	0.656570
x2	1.0	55.125000	55.125000	0.170692	0.707253
x3	1.0	6.125000	6.125000	0.018966	0.899188
x4	1.0	406.125000	406.125000	1.257545	0.343783
x1:x2	1.0	0.125000	0.125000	0.000387	0.985539
x1:x3	1.0	210.125000	210.125000	0.650641	0.478901
x1:x4	1.0	10.125000	10.125000	0.031352	0.870738
x2:x3	1.0	297.874503	297.874503	0.922353	0.407706
x2:x4	1.0	190.477770	190.477770	0.589804	0.498409
x3:x4	1.0	480.500000	480.500000	1.487843	0.309697
Residual	3.0	968.852273	322.950758	NaN	NaN

--- Analyzing Y2 ---

Model Summary (Coefficients and p-values)

OLS Regression Results

==

Dep. Variable: Y2 R-squared: 0.976

Model: OLS Adj. R-squared: 0.921

Method: Least Squares F-statistic: 17.68

Date: Tue, 21 Oct 2025 Prob (F-statistic): 0.0191

Time: 14:53:54 Log-Likelihood: 49.988

No. Observations:	11	AIC:	-83.98
Df Residuals:	3	BIC:	-80.79
Df Model:	7		
Covariance Type:	nonrobust		

==

	coef	std err	t	P>|t|	[0.025	0.975]
Intercept	0.1745	0.001	117.576	0.000	0.170	0.179
x1	1.937e-17	0.002	1.11e-14	1.000	-0.006	0.006
x2	0.0050	0.002	2.872	0.064	-0.001	0.011
x3	0.0150	0.002	8.617	0.003	0.009	0.021
x4	-0.0050	0.002	-2.872	0.064	-0.011	0.001
x1:x2	-4.745e-18	0.001	-5.45e-15	1.000	-0.003	0.003
x1:x3	0.0050	0.001	5.745	0.010	0.002	0.008
x1:x4	2.433e-18	0.001	2.79e-15	1.000	-0.003	0.003
x2:x3	4.167e-18	0.001	4.79e-15	1.000	-0.003	0.003
x2:x4	0.0050	0.001	5.745	0.010	0.002	0.008
x3:x4	-4.745e-18	0.001	-5.45e-15	1.000	-0.003	0.003

==

Omnibus:	2.020	Durbin-Watson:	1.719
Prob(Omnibus):	0.364	Jarque-Bera (JB):	0.207
Skew:	-0.133	Prob(JB):	0.902
Kurtosis:	3.617	Cond. No.	9.85e+16

==

Notes:

[1] Standard Errors assume that the covariance matrix of the errors is correctly specified.

[2] The smallest eigenvalue is 1.65e-33. This might indicate that there are

strong multicollinearity problems or that the design matrix is singular.

ANOVA:

	df	sum_sq	mean_sq	F	PR(>F)
x1	1.0	8.014950e-34	8.014950e-34	3.306167e-29	1.000000
x2	1.0	2.000000e-04	2.000000e-04	8.250000e+00	0.063931
x3	1.0	1.800000e-03	1.800000e-03	7.425000e+01	0.003287
x4	1.0	2.000000e-04	2.000000e-04	8.250000e+00	0.063931
x1:x2	1.0	1.639314e-33	1.639314e-33	6.762171e-29	1.000000
x1:x3	1.0	8.000000e-04	8.000000e-04	3.300000e+01	0.010477
x1:x4	1.0	1.567082e-33	1.567082e-33	6.464214e-29	1.000000
x2:x3	1.0	2.751347e-05	2.751347e-05	1.134931e+00	0.364860
x2:x4	1.0	4.521380e-05	4.521380e-05	1.865069e+00	0.265419
x3:x4	1.0	0.000000e+00	0.000000e+00	0.000000e+00	1.000000
Residual	3.0	7.272727e-05	2.424242e-05	NaN	NaN

```
--- Analyzing Y3 ---
Model Summary (Coefficients and p-values)
                            OLS Regression Results
==============================================================================
Dep. Variable:                     Y3   R-squared:                       0.974
Model:                            OLS   Adj. R-squared:                  0.914
Method:                 Least Squares   F-statistic:                     16.14
Date:                Tue, 21 Oct 2025   Prob (F-statistic):             0.0217
Time:                        14:53:54   Log-Likelihood:                -21.719
No. Observations:                  11   AIC:                             59.44
Df Residuals:                       3   BIC:                             62.62
Df Model:                           7
Covariance Type:            nonrobust
==============================================================================
                 coef    std err          t      P>|t|      [0.025      0.975]
------------------------------------------------------------------------------
Intercept     69.5736      1.006     69.144      0.000      66.371      72.776
```

x1	-0.6050	1.180	-0.513	0.643	-4.360	3.150
x2	2.0650	1.180	1.750	0.178	-1.690	5.820
x3	12.0575	1.180	10.219	0.002	8.303	15.812
x4	-0.0175	1.180	-0.015	0.989	-3.772	3.737
x1:x2	0.9750	0.590	1.653	0.197	-0.902	2.852
x1:x3	0.1362	0.590	0.231	0.832	-1.741	2.014
x1:x4	0.9263	0.590	1.570	0.214	-0.951	2.804
x2:x3	0.9263	0.590	1.570	0.214	-0.951	2.804
x2:x4	0.1363	0.590	0.231	0.832	-1.741	2.014
x3:x4	0.9750	0.590	1.653	0.197	-0.902	2.852

==

Omnibus:	9.522	Durbin-Watson:	0.997
Prob(Omnibus):	0.009	Jarque-Bera (JB):	4.913
Skew:	1.579	Prob(JB):	0.0857
Kurtosis:	3.867	Cond. No.	9.85e+16

==

Notes:

[1] Standard Errors assume that the covariance matrix of the errors is correctly specified.

[2] The smallest eigenvalue is 1.65e-33. This might indicate that there are

strong multicollinearity problems or that the design matrix is singular.

ANOVA:

	df	sum_sq	mean_sq	F	PR(>F)
x1	1.0	2.928200	2.928200	0.262922	0.643488
x2	1.0	34.113800	34.113800	3.063063	0.178396
x3	1.0	1163.066450	1163.066450	104.431232	0.001997
x4	1.0	0.002450	0.002450	0.000220	0.989098
x1:x2	1.0	30.420000	30.420000	2.731399	0.196963
x1:x3	1.0	0.594050	0.594050	0.053339	0.832206

x1:x4	1.0	27.454050	27.454050	2.465087	0.214424
x2:x3	1.0	10.647164	10.647164	0.956004	0.400303
x2:x4	1.0	15.882240	15.882240	1.426059	0.318222
x3:x4	1.0	6.882050	6.882050	0.617936	0.489188
Residual	3.0	33.411455	11.137152	NaN	NaN

--- Analyzing Y4 ---

Model Summary (Coefficients and p-values):

OLS Regression Results

==

Dep. Variable:	Y4	R-squared:	0.952
Model:	OLS	Adj. R-squared:	0.839
Method:	Least Squares	F-statistic:	8.435
Date:	Tue, 21 Oct 2025	Prob (F-statistic):	0.0537
Time:	14:53:54	Log-Likelihood:	-21.976
No. Observations:	11	AIC:	59.95
Df Residuals:	3	BIC:	63.14
Df Model:	7		
Covariance Type:	nonrobust		

==

	coef	std err	t	P>|t|	[0.025	0.975]
Intercept	73.4864	1.030	71.345	0.000	70.208	76.764
x1	1.7312	1.208	1.433	0.247	-2.113	5.575
x2	0.3538	1.208	0.293	0.789	-3.490	4.198
x3	7.6713	1.208	6.351	0.008	3.827	11.515
x4	3.2938	1.208	2.727	0.072	-0.550	7.138
x1:x2	-1.1369	0.604	-1.883	0.156	-3.059	0.785
x1:x3	0.3094	0.604	0.512	0.644	-1.613	2.231
x1:x4	1.3931	0.604	2.307	0.104	-0.529	3.315

x2:x3	1.3931	0.604	2.307	0.104	-0.529	3.315
x2:x4	0.3094	0.604	0.512	0.644	-1.613	2.231
x3:x4	-1.1369	0.604	-1.883	0.156	-3.059	0.785

==

Omnibus:	15.188	Durbin-Watson:	1.299
Prob(Omnibus):	0.001	Jarque-Bera (JB):	8.786
Skew:	-1.924	Prob(JB):	0.0124
Kurtosis:	5.089	Cond. No.	9.85e+16

==

Notes:

[1] Standard Errors assume that the covariance matrix of the errors is correctly specified.

[2] The smallest eigenvalue is 1.65e-33. This might indicate that there are

strong multicollinearity problems or that the design matrix is singular.

ANOVA:

	df	sum_sq	mean_sq	F	PR(>F)
x1	1.0	23.977813	23.977813	2.054609	0.247195
x2	1.0	1.001113	1.001113	0.085783	0.788698
x3	1.0	470.784613	470.784613	40.340557	0.007896
x4	1.0	86.790313	86.790313	7.436882	0.072119
x1:x2	1.0	41.359513	41.359513	3.544011	0.156300
x1:x3	1.0	3.062813	3.062813	0.262446	0.643776
x1:x4	1.0	62.105512	62.105512	5.321693	0.104329
x2:x3	1.0	8.233985	8.233985	0.705553	0.462590
x2:x4	1.0	12.196782	12.196782	1.045117	0.381880
x3:x4	1.0	14.580000	14.580000	1.249330	0.345125
Residual	3.0	35.010767	11.670256	NaN	NaN

Table S4: *In vitro* release profiles of the bioactive compounds (CUR and RESV), expressed as mean ± standard deviation (n=3).

Time (hours)	Curcumin (% mean ± SD)	Resveratrol (% mean ± SD)
1	5.12 ± 0.53	18.22 ± 1.00
3	6.62 ± 0.74	32.22 ± 1.67
6	9.68 ± 2.16	39.11 ± 3.01
9	15.25 ± 0.57	51.12 ± 2.29
12	23.83 ± 1.25	66.06 ± 3.54
24	39.19 ± 9.37	80.47 ± 3.83
48	44.07 ± 3.37	86.76 ± 9.22
72	58.56 ± 2.98	96.95 ± 3.04

Table S5: Comparative analysis of nanocarriers co-encapsulating CUR and RESV

Study	Nanocarrier type	Preparation method	Mean particle size (d.nm)	Drug loading	Stability	Release profile	Key differences vs. present study
Palliyage et al. (2021) [68]	Solid lipid nanoparticles (SLNs)	High-intensity shear homogenization	180.2 ± 7.7	0.49 mg/mL (each)	~2 weeks	~10% CUR / ~51% RESV (120H)	Different lipid system and preparation method; significantly shorter stability
Coradini et al. (2014) [69]	Polymeric nanocapsules with lipid core	Polymer-based encapsulation	~200	0.49 mg/mL (each)	90 days	~35% CUR (72H) / ~90% RESV (24H)	Use of polymeric coating; lower drug loading and limited release
Present study	Nanostructured lipid carriers (NLCs)	High-pressure homogenization	~300	>85 mg/mL (each)	90 days	~59% CUR / ~97% RESV (72H)	Higher co-encapsulation capacity, improved stability, and sustained release for both actives
